# Chronic exposure to MDMA (Ecstasy) elicits behavioral sensitization in rats but fails to induce cross-sensitization to other psychostimulants

**DOI:** 10.1186/1744-9081-2-1

**Published:** 2006-01-04

**Authors:** Gunjan M Modi, Pamela B Yang, Alan C Swann, Nachum Dafny

**Affiliations:** 1 Department of Neurobiology and Anatomy, The University of Texas-Medical School at Houston, P.O. Box 20708, Houston, Texas 77225, USA; 2 Department of Psychiatry and Behavioral Sciences, The University of Texas-Medical School at Houston, P.O. Box 20708, Houston, Texas 77225, USA

## Abstract

**Background:**

The recreational use of 3,4-methylenedioxymethamphetamine (MDMA, ecstasy) among adolescents and young adults has become increasingly prevalent in recent years. While evidence suggests that the long-term consequences of MDMA use include neurodegeneration to serotonergic and, possibly, dopaminergic pathways, little is known about susceptibility, such as behavioral sensitization, to MDMA.

**Methods:**

The objectives of this study were to examine the dose-response characteristics of acute and chronic MDMA administration in rats and to determine whether MDMA elicits behavioral sensitization and whether it cross-sensitizes with amphetamine and methylphenidate. Adult male Sprague-Dawley rats were randomly divided into three MDMA dosage groups (2.5 mg/kg, 5.0 mg/kg, and 10.0 mg/kg) and a saline control group (N = 9/group). All three MDMA groups were treated for six consecutive days, followed by a 5-day washout, and subsequently re-challenged with their respective doses of MDMA (day 13). Rats were then given an additional 25-day washout period, and re-challenged (day 38) with similar MDMA doses as before followed by either 0.6 mg/kg amphetamine or 2.5 mg/kg methylphenidate on the next day (day 39). Open-field locomotor activity was recorded using a computerized automated activity monitoring system.

**Results:**

Acute injection of 2.5 mg/kg MDMA showed no significant difference in locomotor activity from rats given saline (control group), while animals receiving acute 5.0 mg/kg or 10.0 mg/kg MDMA showed significant increases in locomotor activity. Rats treated chronically with 5.0 mg/kg and 10.0 mg/kg MDMA doses exhibited an augmented response, i.e., behavioral sensitization, on experimental day 13 in at least one locomotor index. On experimental day 38, all three MDMA groups demonstrated sensitization to MDMA in at least one locomotor index. Amphetamine and methylphenidate administration to MDMA-sensitized animals did not elicit any significant change in locomotor activity compared to control animals.

**Conclusion:**

MDMA sensitized to its own locomotor activating effects but did not elicit any cross-sensitization with amphetamine or methylphenidate.

## Background

The recreational use of 3,4-methylenedioxymethamphetamine (MDMA, ecstasy) has become increasingly prevalent in recent years, with the highest incidence of use reported in adolescent and young-adult populations [1,2]. An estimated 6.4 million individuals have used MDMA, and its use has risen over the past decade [3]. This high rate of use, especially in younger human populations, is alarming since many animal studies have correlated MDMA use with the onset of lasting cognitive and behavioral deficits, including impairments in spatial memory and learning, increased anxiety-like behavior, and a weakened ability of the nervous system to react to stressful stimuli [4-12], while the addictive potential of MDMA, such as behavioral sensitization, remains poorly understood.

MDMA is a ring-substituted amphetamine derivative with a structural resemblance to the hallucinogen mescaline [13]. Physiological effects of MDMA use include hyperactivity [14-16], hyperthermia [17-20], and hyponatremia [19,21]. Pharmacologically, MDMA potently causes an acute rise in extracellular serotonin (5-HT), which is often behaviorally manifested as "5-HT syndrome" in rats [8,9,14], characterized by low body posture, forepaw treading, and head-weaving. MDMA also causes an acute rise in extracellular dopamine (DA) levels [22,23]. The chronic administration of MDMA has been well-characterized for causing long-term persistent depletions in brain 5-HT levels, most likely due to the neurotoxic action of MDMA to 5-HT-releasing neurons [7,24]. In light of the increased incidence of MDMA use along with data suggesting neurotoxicity and long-term cognitive and behavioral deficits linked to its use, a good understanding of its potential to elicit dependence and abuse is paramount. Behavioral sensitization is one of the experimental markers to indicate the potential of a drug to produce dependence [25-27]. Behavioral sensitization is characterized by a progressively augmented response following repetitive administration of a drug. It is an accepted experimental model to verify for dependency on a drug [25-27]. Although the behavioral sensitization to psychostimulants such as cocaine [27], amphetamine [26,28,29], and methylphenidate [30-32] has been well characterized, the effects of repeated MDMA administration on locomotor activity are contradictory, with some studies suggesting that MDMA elicits behavioral sensitization [18,27,33], and some showing that MDMA causes tolerance [8,34] or neither [16].

Furthermore, behavioral cross-sensitization, which is the augmentation that occurs when pretreatment with one stimulant leads to greater sensitivity to another stimulant, has been established between psychostimulants, including between cocaine and amphetamine [35], methylphenidate and amphetamine [36], and methylphenidate and cocaine [37]. Kalivas et al. [27], Itzhak et al. [38], and Achat-Mendes et al. [39] reported that repeated MDMA treatment prior to cocaine resulted in an elevated behavioral response to cocaine. Pre-exposure to MDMA, therefore, may induce a cross-sensitized response to other psychostimulants such as amphetamine and methylphenidate.

The present study examined the acute and chronic dose-response characteristics on locomotor activity in adult male Sprague-Dawley rats following MDMA and subsequently further re-challenged with amphetamine or methylphenidate. This was done to determine whether MDMA can elicit behavioral sensitization as well as to cross-sensitize with other psychostimulants. A sensitized and/or cross-sensitized response could prove invaluable in determining the potential of MDMA to elicit dependence/addiction [40] and increased susceptibility to abuse of other addictive psychostimulants [27,41].

## Results

### Controls

**Figure 1:**
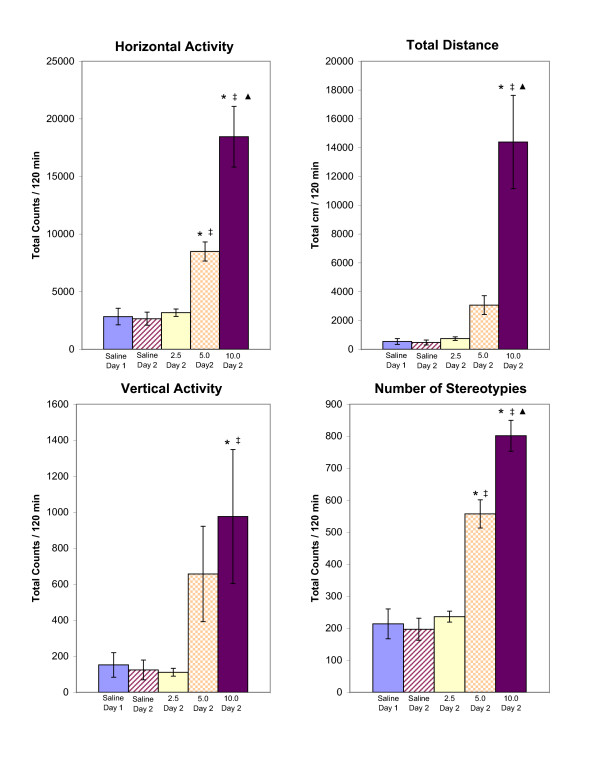
summarizes the experiments measuring acute dose-response to MDMA (2.5, 5.0, and 10.0 mg/kg, i.p.) for the HA, TD, VA, and NOS locomotor indices. The values are presented as the mean ± S.E.M. *, ‡, and ▲ are for significant differences compared to experimental day 2 saline, 2.5 mg/kg MDMA, and 5.0 mg/kg MDMA, respectively (p < 0.05).

In general, saline injections slightly increased locomotor activity during the first 10-minutes post-injection and returned to baseline and remained stable during the entire recording session. Figure 1 shows the activity following 2 consecutive saline injections (experimental days 1 and 2) and demonstrates that activity measured on these two days is similar. Therefore, any changes in locomotor activity recorded that deviate from saline group values obtained on experimental day 2 following MDMA treatment is recognized as an effect of the drug treatment. Previous time control experiments performed by this laboratory on rats given daily saline injections for 42 days have exhibited stable and similar baseline locomotor activity over the course of experiments [42].

### Dose-response effects of acute (single-injection) MDMA administration

The general effects of MDMA were dose-dependent increases in locomotor activity across the indices measured. The lowest dose of MDMA (2.5 mg/kg on experimental day 2), however, showed no effects on any of the locomotor indices studied versus animals given saline. Rats given 5.0 mg/kg MDMA on experimental day 2 (Fig. 1) showed trends of increases in HA, TD, VA and NOS, but only the increases in the HA and NOS indices were significant (p < 0.05) compared to animals given 2.5 mg/kg or saline. Animals injected with 10.0 mg/kg MDMA showed a significant (p < 0.05) increase in all four locomotor indices (HA, TD, VA, and NOS) when compared to the saline or to 2.5 mg/kg MDMA groups. Animals receiving 10.0 mg/kg MDMA also had significantly (p < 0.05) higher activity than animals receiving 5.0 mg/kg across the HA, TD, and NOS indices.

### Dose-response effects of chronic (multiple-injection) MDMA administration

**Figure 2:**
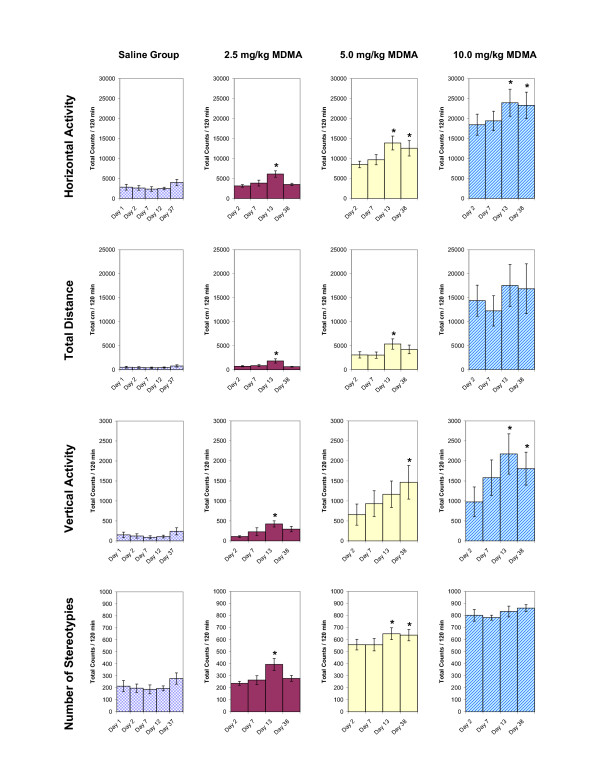
summarizes the experiments measuring the chronic dose-response to MDMA (2.5, 5.0, and 10.0 mg/kg, i.p.) for the HA, TD, VA, and NOS locomotor indices. The values are presented as the mean ± S.E.M. Significance is determined by comparing activity counts to Day 2 (saline day). *p < 0.05.

The total 2-h locomotor activity obtained on experimental days 1, 2, 7, 13 and 38 for the four indices is summarized in figure 2. Saline injections (Fig. 2) had no effect on locomotion and were similar for all the indices (HA, TD, VA, and NOS) studied. The effect of 6 consecutive daily MDMA exhibit similar locomotor activity, i.e. the initial response to MDMA on experimental day 2 was similar to experimental day 7. However, after 5 days of washout (experimental day 13), animals treated with 2.5 mg/kg (i.e., experimental day 13) MDMA showed a significant increase in locomotor activity across all four locomotor indices when compared with experimental day 2. This significant increase in locomotion expresses behavioral sensitization. These same animals, however, showed no significant change in response to 2.5 mg/kg, i.p, MDMA on experimental day 38; i.e., the repetitive treatment of 2.5 mg/kg MDMA elicited sensitization of a transient nature (Fig. 2). Animals treated with 5.0 mg/kg MDMA showed significant increases in locomotor activity on experimental day 13 across the HA, TD, and NOS indices. After 25 days of washout (on experimental day 38), animals re-challenged with 5.0 mg/kg exhibited sensitization across the HA, VA and NOS indices. The locomotor sensitization elicited by the 5.0 mg/kg dose thus persisted for a longer period of time than that of the 2.5 mg/kg dose. In animals injected with 10.0 mg/kg MDMA, the initial high dose elicited a robust increase in locomotor activity, but only the HA and VA indices exhibited sensitization on experimental day 13 and again (25 days later) on experimental day 38 (Figure 2).

The effects of chronic MDMA administration on animal weight and rate of weight gain were not significant compared to animals receiving saline (data not shown).

### Temporal dose-response characteristics of MDMA on locomotor activity

**Figure 3:**
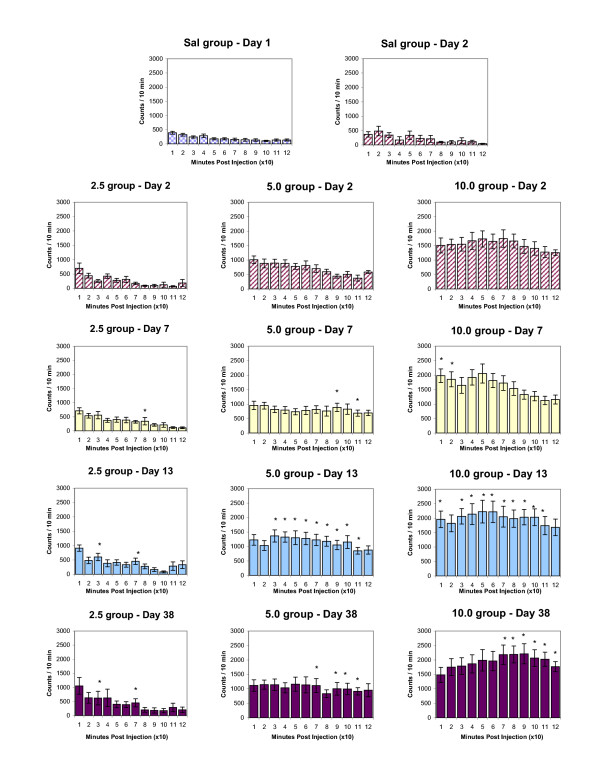
summarizes the temporal pattern of experiments measuring chronic dose-response to MDMA (2.5, 5.0, and 10.0 mg/kg, i.p.) for HA. The values for each 10-minute bin are presented as the mean ± S.E.M. Significance for each bin is determined by comparing activity counts to the same temporal bin on Day 2 (saline day). *p < 0.05.

**Figure 4:**
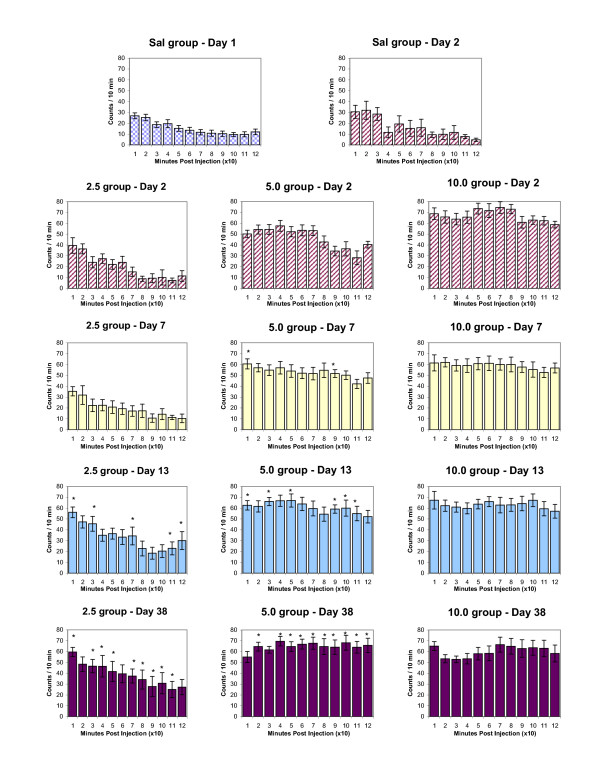
summarizes the temporal pattern of experiments measuring chronic dose-response to MDMA (2.5, 5.0, and 10.0 mg/kg, i.p.) for NOS. The values for each 10-minute bin are presented as the mean ± S.E.M. Significance for each bin is determined by comparing activity counts to the same temporal bin on Day 2 (saline day). *p < 0.05.

Figures 3 and 4 summarize the temporal dose-response characteristics of the HA and NOS indices, on experimental day 2, 7, 13 and 38, respectively. To determine any significant change from baseline values due to drug action, we designated (prior to the experiment) that after drug treatment at least two consecutive 10-minute bins (i.e., significant changes over 20 minutes) with an activity count significantly different (p < 0.05) from the corresponding 10-minute bins were needed in order to characterize the response to the drug as being significantly different from baseline. Based on this criterion, animals injected repeatedly with 2.5 mg/kg MDMA did not express significant change (on experimental days 7, 13, and 38) compared to experimental day 2 in HA (Fig. 3) or TD (data not shown) but showed a significantly increased response in the NOS index on experimental day 13 and experimental day 38 (Fig. 4). Animals injected with 5.0 mg/kg MDMA showed a significant change in the HA and NOS indices in response to MDMA on experimental days 13 and 38, respectively (Figs. 3 and 4). Animals injected with repeated 10.0 mg/kg MDMA showed significant changes in HA (compared to experimental day 2) only in the HA index, seen on experimental days 7, 13, and 38 (Fig. 3). Sensitization was therefore observed and was persistent.

### Interaction between MDMA and amphetamine or methylphenidate

**Figure 5:**
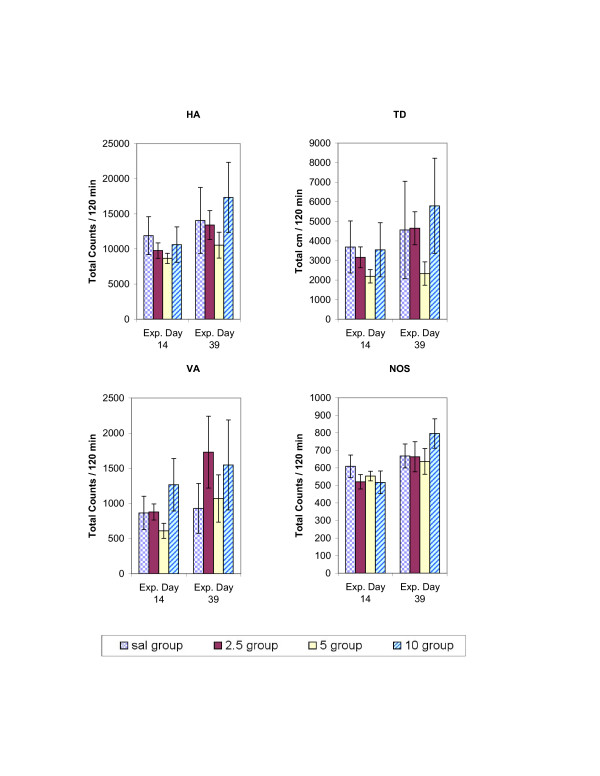
summarizes the effects of a single amphetamine (0.6 mg/kg, i.p.) injection in animals treated chronically with MDMA or saline (see Table 1) for the HA, TD, VA, and NOS locomotor indices on experimental days 14 and 39, comparing activity counts of each MDMA treatment group to the saline group on the same experimental day. The values are presented as the mean ± S.E.M. *p < 0.05.

**Figure 6:**
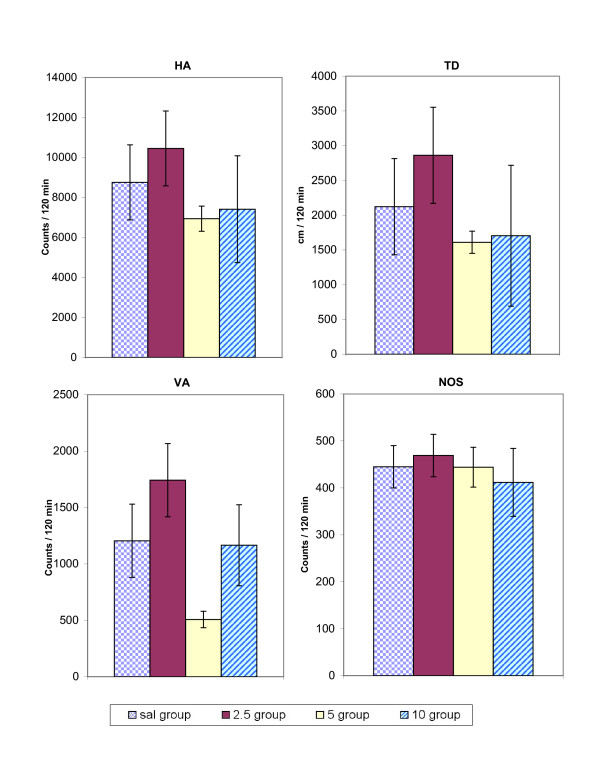
summarizes the effects of a single methylphenidate (2.5 mg/kg, i.p.) injection in animals treated chronically with MDMA or saline (see Table 1) for the HA, TD, VA, and NOS locomotor indices on experimental day 39, comparing activity counts of each MDMA treatment group to the saline group. The values are presented as the mean ± S.E.M. *p < 0.05.

On experimental day 39, six out of nine animals were treated with amphetamine while the remaining three were treated with 2.5 mg/kg methylphenidate (see Table 1 for experimental protocol). The locomotor activity in the saline group resulting from single amphetamine or single methylphenidate injection on experimental day 38 was used as control to compare with the locomotor activity resulting from the same injections in the three MDMA-treatment groups at experimental day 39. This was done to discern any potential interactions such as cross-tolerance or cross-sensitization between MDMA and amphetamine (Fig. 5) or MDMA and methylphenidate (Fig. 6). Figures 5 and 6 summarize this experiment and show that chronic MDMA treatment did not cause any significant changes to locomotor activity when animals were subsequently challenged with either amphetamine or methylphenidate.

**Table 1:** Schedule of Drug Treatment

Experimental Day	Day 1	Days 2–7	Days 8–12	Day 13	Day 14	Days 15–37	Day 37	Day 39
Sal Group (N = 9)	Saline	Saline	Washout	5.0 mg/kg MDMA	Amph	Washout	Saline	Amph/MPD
2.5 Group (N = 9)	Saline	2.5 mg/kg MDMA	Washout	2.5 mg/kg MDMA	Amph	Washout	Saline	Amph/MPD
5.0 Group (N = 9)	Saline	5.0 mg/kg MDMA	Washout	5.0 mg/kg MDMA	Amph	Washout	Saline	Amph/MPD
10.0 Group (N = 9)	Saline	10.0 mg/kg MDMA	Washout	10.0 mg/kg MDMA	Amph	Washout	Saline	Amph/MPD

shows the schedule of drug treatment for the four experimental groups. Locomotor activity was recorded on experimental Days 1–7, 12–14, 37–39. The numbers in the first column (2.5, 5.0, and 10.0) refer to MDMA doses (i.p.). **Amph **= 0.6 mg/kg amphetamine. **Amph/MPD**: On experimental Day 39, animals in each group were given either 2.5 mg/kg methylphenidate (N = 3/group) or 0.6 mg/kg amphetamine (N = 6/group).

## Discussion

The drug MDMA has found increased prevalence in recreational use over the past decade. The question of whether or not it has the potential to elicit dependency is currently the subject of controversy. Several experimental paradigms used to investigate drug dependency include conditioned place preference [15,23,39,43], self-administration [44-47], and behavioral sensitization [14,18,27,38,48,49]. Contradictory results are found whether chronic MDMA administration modulate these three models. Behavioral sensitization is one of the experimental marker that a drug elicits dependence [25,27,36]. The objective of the present study was to use dose-response experiments in an open-field behavioral testing paradigm to investigate whether MDMA will elicit behavioral sensitization and whether chronic MDMA administration results in cross-sensitization to other psychostimulants, namely amphetamine and methylphenidate.

Three MDMA doses were selected for the dose-response study (2.5, 5.0, and 10.0 mg/kg). These doses were chosen based on similar doses used in other studies [14-16,18,38]. Additionally, the MDMA doses administered also fall within the range of MDMA taken recreationally by humans [50]. The amphetamine (0.6 mg/kg) and methylphenidate (2.5 mg/kg) doses were used to assess cross-sensitization because these doses have been established in a previous study to individually elicit behavioral sensitization [29,36].

In general, MDMA caused increases in locomotor activity, and these increases were dose-dependent i.e., increase locomotor activities with increasing the MDMA dose. These findings are similar to those reported by other investigators [43,47,50]. It has been proposed that this locomotor hyperactivity following psychostimulant administration is a result of activation of DA-releasing neurons located primarily in the ventral tegmental area (VTA), nucleus accumbens (NAc), pre-frontal cortex (PFC), and other basal ganglia structures known collectively as the motive circuit [51,52]. However, locomotor hyperactivity induced by MDMA is markedly different from that induced by other psychostimulants such as amphetamine [53,54]. MDMA is believed to activate 5-HT pathways [22,55] and co-activate dopaminergic pathways [50,55-57]. Dose-dependent increases in extracellular dopamine (DA) have been reported in animals following the first few hours post-MDMA administration [50]. Several hypotheses explain how DA is released after MDMA treatment: (1) MDMA interaction with DA uptake carrier causes release of DA [50], (2) the release of DA is the outcome of MDMA entry into DA nerve-ending tissue through diffusion [58], and (3) MDMA elicits DA release through activation of neurons by 5-HT binding of 5-HT_2 _receptor subtypes localized on DA releasing neurons. This last hypothesis is based on the observation that fluoxetine prevented the MDMA-induced release of DA [22,59].

Activation and subsequent reinforcement of primarily dopaminergic pathways in the motive circuit (VTA, NAc, PFC) and basal ganglia is believed to be responsible for the onset of behavioral sensitization due to repetitive (chronic) exposure of drugs, including classically addictive psychostimulants such as cocaine and amphetamine. This motive circuit regions is believed to mediate locomotor activity, and when reinforced, lead to progressive increases in DA and subsequent increase in locomotor activity, characterized as behavioral sensitization [52,60-63]. This behavioral sensitization is used as a marker to indicate the property of a drug to have susceptibility for dependency [25-27].

The present findings indicate that MDMA does indeed elicit behavioral sensitization to its locomotor activating effects similar to other psychostimulants such as cocaine [52], amphetamine [26,28], and methylphenidate [30,31]. The fact that MDMA sensitized to itself indicates that MDMA may have the potential to elicit dependency. Behavioral sensitization following chronic treatment of MDMA using different experimental protocols was reported by some groups [15,18,27,38], and was disputed by other observations [16]. This discrepancy in findings can be partially reconciled by accounting for differences in rat strain, sex, age, and various MDMA treatment protocols used. Investigators who studied only the horizontal activity reported that the drug elicited behavioral sensitization, while those who investigated different motor indices were not able to ascertain whether MDMA elicited sensitization. Specifically, as reported in other studies with psychostimulant sensitization, sensitization tends to be most robust in animals challenged after a sufficiently lengthy washout period following a repeated induction phase of pre-treatment [27,30,31,36,52,63,64]. Moreover, our study analyzed four different motor indices and showed sensitization in two of them and in the other two indices partial sensitization, which may explain this discrepancy, i.e., depending on the motor index used. Therefore, analyzing several locomotor indices is essential.

Cross-sensitization between psychostimulants such as between cocaine and amphetamine [65] and between amphetamine and methylphenidate [36] has been well characterized. Cross-sensitization between two psychostimulants could possibly indicate a similar mechanism in the neural adaptations mediating behavioral sensitization. In this study, animals previously treated with MDMA did not show a cross-sensitized response to amphetamine or methylphenidate. These results suggest that MDMA elicits sensitization by a neural pathway different to that reinforced by chronic amphetamine and methylphenidate. This is validated in cross- self-administration studies performed by Ratzenboeck et al [44]. Similarly, Cole et al. [66] reported that rats pre-treated with MDMA failed to elicit a conditioned place preference to d-amphetamine or cocaine. However, Fone et al. [9], Horan et al. [67], and Achat-Mendes et al. [39] found that rats repeatedly exposed to MDMA in adolescence exhibit an enhanced conditioned place preference to cocaine in adulthood. Morgan et al. [68] reported that repeated MDMA augments cocaine's ability to elevate extracellular DA levels in the rat's nucleus accumbens. Additionally, Fletcher et al., [41] established that repeated pre-exposure to high doses of MDMA facilitates acquisition of cocaine self-administration. Callaway and Geyer [34] reported that pre-treatment of rats with MDMA potentiated the activating effects of d-amphetamine. Inconsistencies in reports investigating cross-sensitization may be explained by the differences in methodologies including (but not limited to) the time and route of drug-administration, age and strain of the animals tested, and experimental paradigms. However, a possible explanation supporting the failure of MDMA to cross sensitize to other stimulants could be given by the presence of evidence suggesting that the primary course of action of MDMA is through 5-HT releasing neurons. While there are reports that MDMA directly impacts DA-releasing neurons, these effects are minor in relation to DA release by 5-HT activation [27]. Blockade of 5-HT_2 _receptor subtypes or serotonin transporters has been demonstrated to be effective in limiting acute DA release by MDMA [22,69]. The lack of a cross-sensitized response to amphetamine and methylphenidate indicates that MDMA may not increase the vulnerability to abuse of other psychostimulants.

## Conclusion

The findings of this study are that (1) an acute dose of MDMA causes dose-dependent increases in locomotor activity; (2) chronic MDMA administration causes transient behavioral sensitization to a low dose (2.5 mg/kg) of MDMA and produces more persistant behavioral sensitization to the higher MPD doses (5.0 and 10.0 mg/kg); (3) chronic MDMA administration did not elicit a cross-sensitized response to amphetamine or methylphenidate; and (4) chronic administration of MDMA did not interrupt animal growth with regard to weight.

## Materials and methods

Thirty-six male Sprague Dawley rats (Harlan, Indianapolis, IN), weighing 200–240 g at the beginning of experiments, were housed in groups of four in Plexiglas cages in an animal housing facility. The facility was maintained on a 12-hour light/dark cycle (lights on at 07:00) with temperature at 21 ± 2°C and the relative humidity at 38%–42%. Water and food pellets were supplied ad libitum. Animals were handled daily in the housing facility for five days prior to experimental manipulation. All experiments were carried out in accordance with the National Institutes of Health Guide for the Care and Use of Laboratory Animals and approved by the University of Texas Center for Laboratory Animal Medicine and Care.

After five days of habituation, animals were taken to a testing room for daily experimentation. The testing room was maintained at the same environmental conditions as the animal housing facility. All experiments were conducted in the testing room between 09:00 and 15:00 h. Each animal was placed individually in a computerized automated activity test chamber that consists of an acrylic open-field box (40.5 × 40.5 × 31.5 cm) equipped with two levels of infrared photo-beam sensors located 6.0 and 12.5 cm above the floor of the box (AccuScan Instruments, Columbus, OH). The system checked for interruptions of each infrared beam at a frequency of 100 Hz. Interruption of any single beam resulted in the recording of an activity count. Simultaneous interruptions of two or more consecutive beams separated by at least one second were recorded as a movement score. This system has been previously described in detail [29,30,32,36].

Locomotor activity counts were summed into 10-minute bins (12 bins per 120-minute session). These data were subsequently downloaded to an IBM-compatible computer through a Versamax analyzer (AccuScan Instruments, Columbus, OH) and sorted into various locomotor indices for analysis. Four locomotor indices were analyzed: horizontal activity (HA), total distance (TD), vertical activity (VA), and number of stereotypies (NOS). The HA index measures the total number of beam interruptions that occurred in the bottom level of infrared beams during a given sample period; TD is a measure of the amount of forward ambulatory activity; VA measures the total number of beam interruptions that occurred in the upper level of infrared beams during a given sample period; and NOS measures the number of repetitive episodes with at least a 1-second interval before the beginning of another episode. The NOS index is used to assess the effect of drug treatment on general stereotypic behaviors such as sniffing, grooming, and other repetitive behaviors.

On each testing day, animals were placed in the open-field chambers and habituated for 30 minutes, after which injections were administered and subsequent locomotor activity was recorded for 120 minutes. On experimental day 1 (see Table I), all animals were injected with saline prior to recording activity. Beginning with experimental day 2, rats were divided into four experimental groups (N = 9/group) consisting of three MDMA-treatment groups (2.5, 5.0, and 10.0 mg/kg) and one control (saline) group. On experimental days 2–7, animals were treated in their testing cages with their respective doses of MDMA, and animals in the control group were given saline. All animals were then given 5 days of washout (experimental days 8–12). On experimental day 13, animals were re-challenged with the same respective doses of MDMA given during the initial 6 days of treatment. Days 14–37, no injections were given, activity was not monitored, and animals were kept in the housing facility. On experimental day 38, animal activity was recorded again, following MDMA rechallenge as in experimental day 13 (Table 1). On experimental day 39, six animals challenged with 0.6 mg/kg amphetamine and three animals with 2.5 mg/kg, i.p., methylphenidate (Table 1). The drug treatment paradigm was adapted from similar psychostimulant dose-response studies previously conducted by our laboratory [28,29,31,32,36]. Upon completion of daily locomotor activity recordings and injection, animals were returned to the housing facility.

All doses of (±)-3,4-methylenedioxymethamphetamine hydrochloride (MDMA; NIDA, Research Triangle Park, NC), d-amphetamine sulfate (Sigma-Aldrich, St. Louis, MO), and methylphenidate hydrochloride (Mallinckrodt, St. Louis, MO) were calculated as free base and dissolved in 0.9% saline for administration. Injections were all administered intraperitoneally (i.p.) and equalized with 0.9% saline to a volume of 0.8 ml so that the total volume of injection would not vary between animals.

The acute locomotor effects of MDMA were evaluated by comparing all three MDMA-treated groups on experimental day 2 against the saline-treated group on experimental day 1. Sensitization to MDMA was determined by comparing activity scores from initial MDMA treatment (experimental day 2) with the activity scores recorded on the last day of MDMA maintenance (experimental day 7) and MDMA re-challenge days (experimental days 13 and 38). The presence of MDMA cross-sensitization to amphetamine or methylphenidate was determined between treatment groups by comparing the activity response to amphetamine challenge (experimental days 39) and methylphenidate challenge (experimental day 39) of the saline group (control group; animals pre-treated with saline) with the activity response to amphetamine/methylphenidate challenge of the MDMA-treated groups. Observations within groups were analyzed using one-way Analysis of Variance (ANOVA: treatment days) and post-hoc Fischer's LSD method. Observations between groups were compared using the Student's paired t-test. Statistical significance was set at p < 0.05 for all comparisons.

## Authors' contributions

The experiment was conceived, developed, and reported collaboratively by all authors. GMM was primarily responsible for data collection and analysis.
